# Argyrophilic nucleolar organizer regions (AgNOR) in gastric cell of obese patients with bariatric surgery

**DOI:** 10.1097/MD.0000000000045278

**Published:** 2025-10-10

**Authors:** Zerrin Gamsizkan, Sinem Kantarcioğlu Coşkun, Recep Eröz, Mevlüt Pehlivan

**Affiliations:** a Department of Family Medicine, Duzce University Medical Faculty, Duzce, Turkey; b Department of Pathology, Duzce University Medical Faculty, Duzce, Turkey; c Department of Medical Genetics, Aksaray University Medical Faculty, Aksaray, Turkey; d Department of General Surgery, Duzce University Medical Faculty, Duzce, Turkey.

**Keywords:** AgNOR staining, NOR protein, obesity, obesity etiology, obesity management

## Abstract

The etiology of obesity, which is considered a pandemic, is influenced by several factors. According to the literature, gastric cells in obese patients have not been examined by silver-stained nuclueolus organizing regions (AgNOR) staining. This study investigated the relationship between obesity, nucleolus organizing region (NOR) protein synthesis, and histopathological findings. Nonobese patients undergoing gastric biopsy for various reasons and patients undergoing bariatric surgery were included. Histopathological findings, mean AgNOR count, and total AgNOR area/nuclear area ratio (TAA/NA) were evaluated for each case. In the study, 30 nonobese patients who underwent gastric biopsy and 28 patients who underwent bariatric surgery were included. Additionally, no significant differences were found in the TAA/NA ratios with respect to the presence or absence of histological activity, intestinal metaplasia, or *Helicobacter pylori* infection (*P* = .686, *P* = .588, and *P* = .069, respectively). However, a statistically significant difference in the TAA/NA ratio was observed between the patients with and without lymphocytic infiltration (*P* < .013). This study suggests that NOR protein synthesis is associated with lymphocytic infiltration in patients with treatment-resistant obesity. These findings may contribute to a better understanding of the underlying etiology of obesity, and should be supported by further comprehensive studies.

## 1. Introduction

Obesity has long been recognized as a global epidemic. The global prevalence of obesity has tripled over the past 40 years, reaching alarming levels globally. It is widely acknowledged that addressing the obesity epidemic is complex due to the involvement of numerous contributing factors.^[1]^ Various studies have shown that genetic, chronobiological, and environmental factors play significant roles in the etiology of obesity.^[2–4]^

Although there are many treatment strategies aligned with the World Health Organization’s recommendations to reduce the burden of obesity, relapses frequently occur in the long term.^[5]^ Therefore, genetic and cellular-level studies are essential, especially those focusing on resistant obesity, to improve treatment effectiveness and prevent recurrence.

The organization of the nuclei within cells often displays distinct characteristics in various diseases. Nucleolus organizing regions (NORs) are genetic regions located on the short arms of acrocentric chromosomes that consist of ribosomal DNA (rDNA) and proteins, some of which are argyrophilic. In human interphase cells, silver-stained NOR clusters appear within the nucleolus and represent the sites of ribosomal RNA synthesis and transcriptionally active NORs.^[6]^

These silver-binding proteins are known as argyrophilic NOR (AgNOR)-associated proteins. Silver staining is considered the most reliable technique for visualizing nucleoli in the interphase nuclei. The examination of cell nuclei using AgNOR staining offers valuable insights into the pathogenesis of many diseases. The AgNOR method involves staining non-histone proteins (AgNOR proteins) found in ribosomal DNA regions, responsible for rRNA synthesis, so they appear as black or silver particles.^[7]^ Detection of AgNOR proteins has contributed to the understanding of the etiopathogenesis of various conditions, including cancers,^[8–10]^ Xeroderma Pigmentosum,^[11]^ salivary gland disease_,_^[12]^ and hypoxic injury.^[13,14]^

In studies of AgNOR in various disorders of gastric cells, the number of AgNORs per nucleus was correlated with the proliferative rate.^[15,16]^ It has been reported that, as in other cancers, the shape and number of NORs in gastric cancer show significant variability. NORs appear to reflect the proliferative activity of the cells.^[17,18]^ The results of such studies suggest that NOR reflects the proliferative activity of cells. However, no study has been conducted on AGNOR activity in the gastric mucosa of obese patients. As is well known, obesity activates inflammatory processes. Changes in cell nuclei may also be observed in the gastric mucosa of obese patients.

It is well known that continued weight gain following bariatric surgery suggests that the etiology of obesity is not solely mechanical. This highlights the need for a detailed investigation of additional contributing factors. Previous studies have shown that hormonal and psychological variables can influence weight loss outcomes after laparoscopic sleeve gastrectomy. However, other potential contributors may include histopathological abnormalities in the stomach, which could affect the production of ghrelin and other enterohormones.^[19]^

The aim of this study is to investigate whether there are any differences in NORs in the gastric cells of obese and normal-weight patients.

## 2. Materials and methods

### 2.1. Study design and population

The study was planned as a retrospective and cross-sectional study and included nonobese patients who underwent stomach biopsy for various reasons, and obese patients who underwent bariatric surgery. Gastric cell samples from patients who underwent bariatric surgery to treat obesity over a 1-year period were compared with cells from a similar number of nonobese, randomly selected patients who underwent gastric biopsy. This study was approved by the Ethics Committee of the Duzce University (2020/136).

### 2.2. AgNOR staining

**Figure 1.**
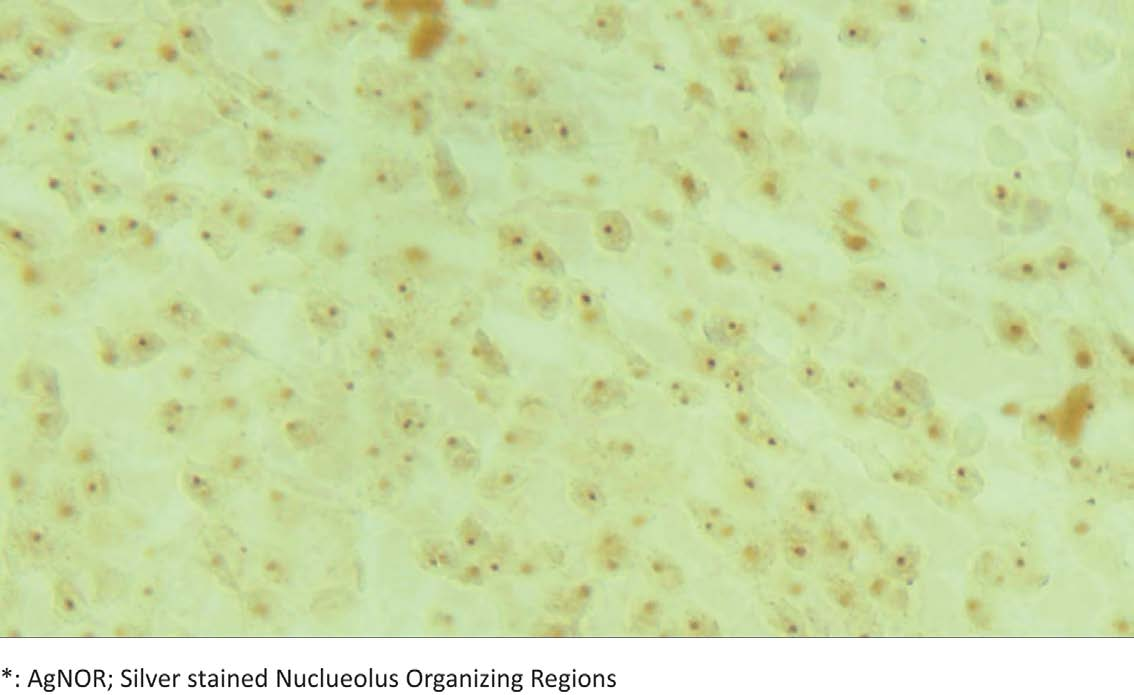
Evaluation AgNOR staining of stomach cells. AgNOR = silver-stained nuclueolus organizing regions.

Slides with a thickness of 4 μm were prepared from paraffin-embedded stomach biopsy tissues from both the groups. The tissue sections were deparaffinized in xylene, rehydrated in graded alcohol solutions, and air-dried at room temperature. The slides, including stomach biopsy materials of both groups, were stained with silver according to the Benn, Perle, and Lindner protocol, with slight modifications.^[20,21]^ In this study, the AgNOR-staining properties of gastric cells of the obese and control group patients were evaluated (Fig. 1).

### 2.3. Image analysis of mean AgNOR Number and total AgNOR area/total nuclear area (TAA/TNA) ratio

Fifty silver-stained nuclei from each slide were photographed using a light microscope (Eclipse 80i; Nikon, Tokyo, Japan) attached to a digital camera (Digital Sight DS-Fi1c; Nikon) and evaluated using ImageJ version 1.47t image processing software (Rasband WS. ImageJ; U.S. National Institutes of Health, Bethesda; 1997–2016). For the detection of both the total AgNOR area-to nuclear area ratio and the mean AgNOR number using “‘freehand selection’” tool for each nucleus.

### 2.4. Statistical method

Statistical analysis was performed using the Statistical Package for Social Sciences (SPSS 22). Descriptive statistical methods, Fisher exact test, and Student *t* test were used to compare groups. Data are given as the mean ± SD, and statistical significance was set at *P* < .05.

## 3. Results

This study included 30 nonobese patients who underwent stomach biopsy for various reasons, and 28 patients who underwent bariatric surgery. Of the patients included in the study, 22 were male and 36 were female, and the mean age was determined as 43.18 ± 14.80. 75% (n = 21) of patients who underwent bariatric surgery had gastritis pathology in their stomach.

In the study, there was no significant difference between the “Total AgNOR area/nuclear area” ratios in the stomach cells of patients with and without the presence or absence of activity, intestinal metaplasia, *H. pylori (P* = .686, *P* = .588, *P* = .069). However, a significant difference was detected between the “Total AgNOR area/nuclear area” ratios in the stomach cells of patients with and without lymphocystic infiltration (*P* < .013, Table 1).

**Table 1:** Patient characteristics and AgNOR* staining.

	N	TAA/NA†	*P*	NOR	*P*
Lymphocytic infiltration					
Yes	45	25.94	.245	1.16	.013
No	13	23.36		1.12	
Activity					
Yes	15	23.74	.277	1.16	.686
No	43	25.93		1.16	
Intestinal metaplasia					
Yes	7	30	.02	1.17	.588
No	51	24.73		1.16	
*H. pylori*					
Yes	32	24.01	.107	1.17	.069
No	26	27.03		1.14	

* Silver-stained nuclueolus organizing regions.

† TAA/NA = total AgNOR area/nuclear area ratio.

**Figure 2.**
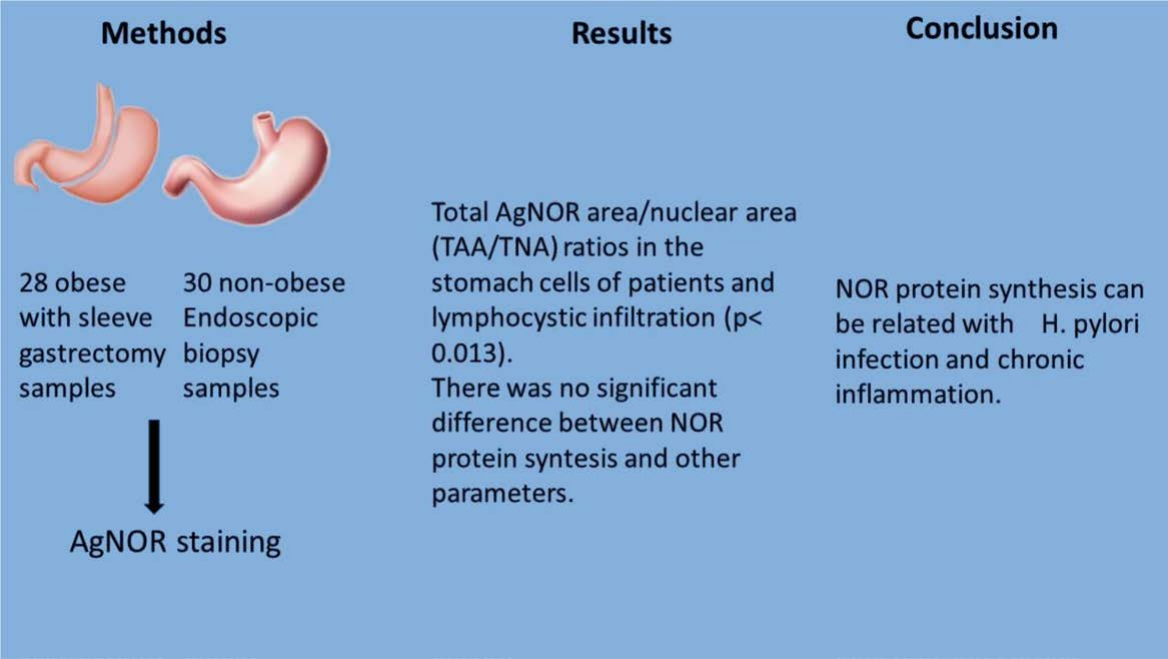
Research design and highlights. AgNOR = silver-stained nuclueolus organizing regions, NOR = NORs = nucleolus organizing regions, TAA/NA = total AgNOR area/nuclear area ratio.

There was no significant difference between the relapse cases and the nonreccurent cases in terms of “Total AgNOR area/nuclear area” and mean AgNOR number (*P* = .684, *P* = 747). There was no significant difference between the relapse cases and the nonreccurent cases the presence or absence of activity, intestinal metaplasia, *H. pylori* and ratios (*P* = .553, *P* = .530, *P* = .062, Table 2, Fig. 2).

**Table 2:** Clinicopathological features of patients based on relapse.

	Relapse		
	Yes	No	*P*
TAA/NA*	25.26	26.79	.684
Mean AgNOR† number	1.11	1.12	.747
Lymphocytic infiltration			
Yes	5	1	.069
No	8	14	
Activity			
Yes	6	0	.553
No	17	5	
Intestinal metaplasia			
Yes	5	1	.530
No	20	2	
*H. pylori*			
Yes	6	0	.062
No	12	10	

* TAA/NA; total AgNOR area/nuclear area ratio.

† AgNOR = silver-stained nuclueolus organizing regions.

## 4. Discussion

In our study, the etiology of obesity was investigated by examining the cells of patients with obesity using silver staining. In our study, a significant relationship was found between the number of NORs of lymphocytic infiltration in gastritis and the ratio of total NOR area to core area. Literature shows that AgNOR examinations have not been performed in obese patients; however, the relationship between histopathological examinations and obesity surgery results has been investigated. In their study, Taskinuresin et al suggested that weight loss and histopathological changes after bariatric surgery were associated with morbidly obese patients.^[22]^ Another study examining the relationship between the histopathological findings of stomach samples and weight loss at 6- and 12-month follow-ups after laparoscopic sleeve gastrectomy surgery also pointed out that there may be a relationship between weight loss after bariatric surgery and stomach histopathology.^[23]^

We also evaluated whether lymphocytic infiltration was associated with recurrence and weight gain after bariatric surgery. Although we did not find significance, we observed increased lymphocytic infiltration in nearly all recurrent cases. The significantly different lymphocytic patterns observed in patients with recurrence in our study suggest that future research should focus on the histopathological factors.

In addition, most of the patients who underwent bariatric surgery in our study had gastritis pathology in their stomachs. Histopathological studies have indicated that chronic gastritis may play a role in the development of severe obesity.^[24,25]^ Obesity-induced metabolic dysfunction may directly influence T-cell responses, further supporting this perspective.^[26]^ Therefore, larger-scale studies are warranted to explore lymphocytic changes in the gastric mucosa of patients with treatment-resistant obesity.

### 4.1. Strengths and limitations

To the best of our knowledge, no previous study has applied AgNOR staining to gastric tissues of obese patients. This study makes a novel contribution to the literature as a first investigation of its kind. However, this study has some limitations. Owing to the small number of patients with recurrence, these findings cannot be generalized to the entire obese population. Further studies with larger cohorts are required to validate these results.

## 5. Conclusion

Although an increase in NOR protein synthesis in treatment-resistant obese patients could not be conclusively demonstrated owing to the limited sample size, our findings suggest a potential association between NOR protein expression and lymphocytic infiltration. Future studies with larger sample sizes are needed to further investigate this relationship and may provide valuable insights into the underlying etiology of obesity.

## Author contributions

**Data curation:** Zerrin Gamsizkan, Sinem Kantarcioglu Coskun, Mevlüt Pehlivan.

**Formal analysis:** Zerrin Gamsizkan, Recep Eröz.

**Methodology:** Zerrin Gamsizkan, Sinem Kantarcioglu Coskun, Recep Eröz.

**Supervision:** Mevlüt Pehlivan, Recep Eröz.

**Writing – original draft:** Zerrin Gamsizkan, Sinem Kantarcioglu Coskun, Recep Eröz, Mevlüt Pehlivan.
